# Diesel engine exhaust accelerates plaque formation in a mouse model of Alzheimer’s disease

**DOI:** 10.1186/s12989-017-0213-5

**Published:** 2017-08-30

**Authors:** Maja Hullmann, Catrin Albrecht, Damiën van Berlo, Miriam E. Gerlofs-Nijland, Tina Wahle, Agnes W. Boots, Jean Krutmann, Flemming R. Cassee, Thomas A. Bayer, Roel P. F. Schins

**Affiliations:** 1 IUF - Leibniz Research Institute for Environmental Medicine, Auf’m Hennekamp 50, 40225 Düsseldorf, Germany; 2 National Institute for Public Health and the Environment, Bilthoven, The Netherlands; 3 Department of Pharmacology and Toxicology, NUTRIM School of Nutrition and Translational Research in Metabolism, Maastricht University, Maastricht, The Netherlands; 4 Medical Faculty, Heinrich-Heine-University, Düsseldorf, Germany; 5 Institute of Risk Assessment Sciences, Utrecht University, Utrecht, The Netherlands; 6 Department of Psychiatry and Psychotherapy, Division of Molecular Psychiatry, Georg-August-University Göttingen, University Medicine Göttingen, Göttingen, Germany; 7 Present address: Triskelion BV Utrechtseweg 48, 3704 HE Zeist, The Netherlands

**Keywords:** Diesel engine exhaust, Inhalation, Particulate matter, 5XFAD mice, Alzheimer’s disease, Amyloid-β, Behaviour

## Abstract

**Background:**

Increasing evidence from toxicological and epidemiological studies indicates that the central nervous system is an important target for ambient air pollutants. We have investigated whether long-term inhalation exposure to diesel engine exhaust (DEE), a dominant contributor to particulate air pollution in urban environments, can aggravate Alzheimer’s Disease (AD)-like effects in female 5X Familial AD (5XFAD) mice and their wild-type female littermates. Following 3 and 13 weeks exposures to diluted DEE (0.95 mg/m^3^, 6 h/day, 5 days/week) or clean air (controls) behaviour tests were performed and amyloid-β (Aβ) plaque formation, pulmonary histopathology and systemic inflammation were evaluated.

**Results:**

In a string suspension task, assessing for grip strength and motor coordination, 13 weeks exposed 5XFAD mice performed significantly less than the 5XFAD controls. Spatial working memory deficits, assessed by Y-maze and X-maze tasks, were not observed in association with the DEE exposures. Brains of the 3 weeks DEE-exposed 5XFAD mice showed significantly higher cortical Aβ plaque load and higher whole brain homogenate Aβ42 levels than the clean air-exposed 5XFAD littermate controls. After the 13 weeks exposures, with increasing age and progression of the AD-phenotype of the 5XFAD mice, DEE-related differences in amyloid pathology were no longer present. Immunohistochemical evaluation of lungs of the mice revealed no obvious genetic background-related differences in tissue structure, and the DEE exposure did not cause histopathological changes in the mice of both backgrounds. Luminex analysis of plasma cytokines demonstrated absence of sustained systemic inflammation upon DEE exposure.

**Conclusions:**

Inhalation exposure to DEE causes accelerated plaque formation and motor function impairment in 5XFAD transgenic mice. Our study provides further support that the brain is a relevant target for the effects of inhaled DEE and suggests that long-term exposure to this ubiquitous air pollution mixture may promote the development of Alzheimer’s disease.

## Background

An increasing number of studies indicates that chronic exposure to ambient particulate matter (PM) may have toxic effects on the nervous system. In urban environments, diesel engine exhaust (DEE) emissions represent a dominant source of the particulate fraction of air pollution [1]. Inhalation toxicology studies in rodents have shown that markers of neuroinflammation and neurotoxicity are induced upon exposures to PM [2–4] and DEE [5–9]. These experimental findings are in line with a growing number of epidemiological studies that demonstrated associations between exposure to PM or traffic-related air pollution and cognitive function or cognitive decline [10–16].

Meanwhile, the specific concern has risen that long-term exposure to particulate air pollution could contribute to the pathogenesis of Alzheimer’s disease (AD) [17–19]. AD is the most common cause of dementia worldwide affecting millions of people. AD is clinically characterised by progressive loss of memory associated with cognitive deficits extending to language skills, decision-making ability, movement and recognition [20]. Neuropathologically, this disease is characterised by the presence of neurofibrillary tangles, intracellular aggregates consisting of hyperphosphorylated Tau proteins and extracellular amyloid plaques, associated with widespread loss of neurons in brain [21]. A major component of the amyloid plaques is the hydrophobic 4kD Amyloid-β peptide (Aβ), which is generated by sequential proteolysis of the amyloid precursor protein (APP) by β- and γ-secretase (reviewed in [22]). The β-secretase cuts APP at the N terminus of the Aβ domain and subsequent cleavage by the γ-secretase at the C terminus of Aβ domain generates a series of Aβ peptides of 38–43 amino acids in length [23]. A strong genetic association between early onset familial forms of AD (FAD) and the 42 amino acid Aβ species (Aβ42) has been demonstrated (reviewed in [24]). FAD-related autosomal dominant mutations in the genes for Presenilin (PS) 1 and 2, subunits of the γ-secretase complex, or in the gene for APP are known to elevate the production of Aβ42 [22]. As Aβ42 is more fibrillogenic than shorter Aβ peptides, increased levels of Aβ42 are thought to drive the formation of the insoluble fibrils that compose amyloid plaques [25].

The earliest clues for a link between (particulate) air pollution exposure and AD came from studies from Calderón-Garcidueñas et al. [26] who compared brain autopsies from lifelong residents from cities with severe air pollution versus cities with low levels of air pollution [26, 27]. Besides markers of inflammation and DNA damage, they found augmented Aβ42 protein levels in frontal cortex, hippocampus and olfactory bulb among the individuals from the highly polluted cities. More recently, they also showed increased presence of markers of oxidative stress, inflammation and neurodegeneration as well as Aβ diffuse plaques in brain autopsies of children and young adults from highly polluted regions. This led the authors to suggest that air pollution plays a role in central nervous system (CNS) damage at a young age with potential development of AD [28]. Experimental clues have become available from Levesque et al. [8] who detected enhanced Aβ42 levels in brains of rats exposed to DEE and Bhatt et al. [29] who showed increased levels of Aβ40 and β-secretase in mice exposed to concentrated PM. The effect of air pollution and dementia is further supported by a recent critical systemic review [30] and outcomes from a major population-based neurological diseases cohort study, showing an association of residential proximity to major roadways with dementia among 243,611 incident cases [16]. Regarding observed epidemiological associations between PM and AD, it has been stated that: “If these data reflect causality, PM exposure would be one of few AD risk factors that are not only widespread, but that also can be modified at the population level using regulatory intervention” [18].

Whether and how long-term exposure to air pollution particles could contribute to pathogenesis of AD is not clear yet. Therefore, we performed a sub-chronic whole-body DEE inhalation study in female transgenic 5X Familial Alzheimer’s Disease (5XFAD) mice and their female wildtype (WT) littermate controls. We hypothesized that DEE could accelerate AD-like effects in this transgenic mouse model. Following exposures for 3 or 13 weeks to DEE or clean air (controls), the mice were subjected to behaviour tests, and then analysed for Aβ plaque formation in hippocampus and cortex. To evaluate the potential involvement of peripheral inflammation, which has been discussed to be involved in AD-pathogenesis [31–33], the mice were also evaluated for the presence of pulmonary histopathology and markers of systemic inflammation.

## Methods

### Animals

Female 5XFAD transgenic mice were used as a model for AD. These mice overexpress the 695 amino acid isoform of the human amyloid precursor protein (APP695) carrying Swedish (K670 N), London (V717I) and Florida (I716V) mutations as well as the human PS1 (M146 L; L286 V) mutations [34, 35]. The mice develop a specific phenotype showing high APP expression levels, amyloid deposition beginning with two month of age and memory impairments and motor deficits [34, 36]. Breeding was performed by mating heterozygote transgenic founders with C57Bl/6 J wild-type (WT) mice to obtain littermate controls for the experiments. Only female mice were used for the study in view of the reported sex-specific differences in age- and treatment related Aβ development [37]. The mice were handled according to guidelines of the Society for Laboratory Animals Science (GV-SOLAS). The study was approved by the Animal Ethics Committee (IUCAC) of the Dutch National Vaccine Institute (NVI, Bilthoven, Netherlands) (project number 201000169). All animals included in this study were born within a time range of 4 days, and age was 10 weeks when inhalation exposures started. Temperature and the relative humidity in the inhalation units [38] were controlled at 22 ± 2 °C and at 40–70%, respectively. Lighting was artificial with a sequence of 12 h light (06:00–18:00) and 12 h dark. Commercially available rodent food pellets and water were provided ad libitum.

### Diesel engine exhaust (DEE) exposure and characterisation of the test atmosphere

**Fig. 1:**
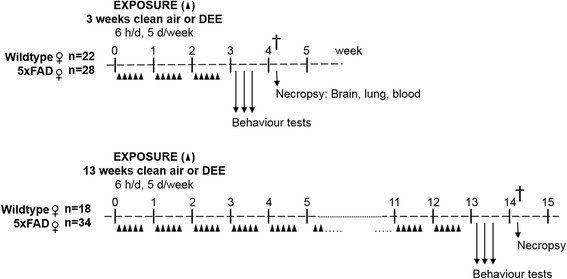
Study design. Female 5X Familial AD (5XFAD) mice and wild-type (WT) female littermates were exposed for 3 or 13 weeks to clean air or diluted diesel engine exhaust (DEE). Animals were born within a time range of 4 days, and age was 10 weeks at exposure start

All animals were exposed in whole body inhalation chambers in separate inhalation units (2 mice per cage, and a maximum of 10 per unit) for 5 days/week, for 6 h a day during either 3 or 13 weeks. The design of the study is depicted in Fig. 1. Animals were exposed to control (conditioned, purified and HEPA-filtered) air or to DEE diluted by mixing the freshly generated exhaust from a stationary diesel engine (Common-rail motor, 100 kVA, 35 KW load) with conditioned purified air. Particle number and mass concentrations were determined continuously using a condensation particle counter (CPC model 3022A, TSI St. Paul, MN, USA), and a Tapered Element Oscillating Microbalance (TEOM 1400a, Rupprecht & Patashnick, Albany, NY, USA), respectively. The particle size distribution was measured 3–4 times during exposure by a Scanning Mobility particle Sizer (SMPS 3080 with CPC 3788, TSI Inc. Shoreview, MN, USA) Time-integrated particle concentrations were analyzed by gravimetric analyses using particles collected on 47 mm Teflon filters. Carbon monoxide and nitrogen oxides were measured continuously by a Gas Filter Correlation CO analyzer (Thermo Electron instr Model 48, Madison, WI, USA) and a Chemiluminescense NO/NOx analyzer (Advancend Pollution Instruments Model 200E, San Diego, Ca, USA). Exposure characteristics are shown in Table 1.

**Table 1:** Exposure characteristics based on measurements in three inhalation units

Parameter	Value
Mass concentration (mg/m^3^)^a^ [SD]	0.95 [5–12%^b^]
Number concentration^c^ (#/cm^3^) [SD]	2.1 • 10^6^ [0.35 • 10^6^]
Geometric mean size (nm), [geometric SD (nm)]	82 [1.75]
CO (ppm) [SD]	6.37 [1.37]
NO (ppm) [SD]	23.11 [5.50]
NO_2_ (ppm) [SD]	1.56 [0.40]
NO_x_ (ppm) [SD]	24.59 [5.81]

^a^Time integrated Filter; ^b^depending on the exposure unit; ^c^CPC; *SD* standard deviation

### Behaviour tests

The behaviour tests were performed during the week after the last exposure, starting on the third day after the last exposure day (see Fig. 1). Motor coordination and grip strength of the mice were tested using a string suspension task. Therefore, a 2 mm broad and 35 cm long cotton string was stretched between two vertical poles above a cushioned bottom. The animals were carried by their tails and permitted to grasp the string by their forepaws before their release. A rating system from 0 to 5 was assigned to each animal during a single 60 s session [39], as follows: 0, unable to hang on the string; 1, hangs only by forepaws; 2, attempting to climb the string; 3, climbing the string with four paws successfully; 4, moving laterally along the string; 5, escaping to one of the platforms at the end of the string. The Y-maze and Cross (X)-maze tasks were applied to reflect spatial working memory of mice by spontaneous alternation, based on the natural affinity of mice to explore a novel environment [40]. For both mazes, alternation percentages were calculated as % of the actual alternations to the possible arm entries. To avoid influence on the behaviour of the mice due to odour of mice tested before, the mazes were thoroughly cleaned after every trial with 70% ethanol.

### Necropsy

Eleven days after the last exposure to the DEE or HEPA-filtered air, the mice were sacrificed by cervical dislocation and heparin-blood was immediately collected. After centrifugation at 2000 g, the plasma was stored at −80 °C until further analysis. Brains were carefully removed after opening of the scull and dissected. The left hemispheres were stored for immunohistochemical analyses after fixation with 4% paraformaldehyde at 4 °C and subsequent embedding in paraffin. The right hemispheres were snapfrozen for later analyses. Lungs were used for immunohistochemical analyses after perfusion and fixation in 10% formalin or dissected, followed by snapfreezing.

### Immunohistochemical analyses of paraffin embedded slices

After sacrificing the animals and careful dissection of their brains, the right half of the brain was fixated in 4% buffered formalin at 4 °C for a minimum of 24 h. Dehydration was performed in a series of ethanol and followed by a transfer into xylene. Afterwards brains were embedded in paraffin. Four μm thick paraffin sections were cut using a sliding microtome, transferred on Superfrost Ultra Plus object slides and dried over night at 40 °C. Subsequently, sections were deparaffinised in xylene, followed by a rehydration in a series of ethanol (100%, 96%, 70%) and blocking of endogenous peroxidase by a treatment with 0.3% H_2_O_2_ in PBS. Antigen retrieval was performed by boiling slices in 10 mM citrate buffer, pH 6.0 followed by an incubation for 3 min in 88% formic acid. Unspecific antibody binding was blocked, via incubation in 10% fetal calf serum (FCS) and 4% skim milk in 0.01 M PBS before application of the primary anti human Aβ antibody (Merck Millipore, Darmstadt, Germany) diluted 1:1000 in 0.01 M PBS and 10% FCS, overnight in a humid chamber at room temperature. After washing slices were incubated with a biotinylated anti-mouse secondary antibody diluted 1:200 in 0.01 M PBS and 10% FCS, and the signal was detected via avidin-biotin-complex-method (ABC) by a Vectastain kit (Vectorlabs, Burlingame, USA) using diaminobenzidine (DAB, Sigma-Aldrich, Deisenhofen, Germany)) as chromogen and Hematoxylin for nuclear counterstaining. Light microscopical pictures from cortex and hippocampus were taken with 100 x or 50 x magnification, respectively, using a Zeiss Axiophot microscope equipped with AxioCam MRc (Carl Zeiss, Jena, Germany). Quantitative Aβ42 plaque analyses were performed via calculation of the % of total plaque load in the analysed area of the section. Plaque load was determined using ZEN 2011 image processing software (Zeiss) after a fixed adjustment of contrast threshold for stained Aβ42 plaques. In detail, the brain section was placed under the microscope at a magnification of 50×. For the analysis of the hippocampal area, this specific part was positioned in the middle of the focus and the region of interest was marked interactively for each animal. After analysis of the hippocampal region, the slide was shifted to a frontal cortex region, which was comparable for each animal. The objective was changed to a magnification of 100× and the complete image section was analysed. From each animal three brain slides with an interspace of approximately 30 μm were analysed and calculated as the % of total plaque load in the analysed area of the section.

### Amyloid-β42 ELISA

Aβ40 and Aβ42 are the two major isoforms of amyloid-β. It has been shown that the transgenic 5XFAD mice produce Aβ42 in a highly predominant manner [34]. Thus, we determined only the levels of this isoform in the brains. The human Aβ42 ELISA was performed according to the manufacturer’s manual (Thermo Fisher Scientific, Darmstadt, Germany). Therefore, frozen brain tissues were homogenised in a potter tissue grinder in 1500 μl Aβ42-ELISA homogenisation buffer (5 M guanidine HCl, 50 mM Tris HCl, pH 8.0), centrifuged for 15 min at 14,000 x g and supernatant was aliquoted and stored at −20 °C until further analysis. For the ELISA measurements, a 96-microtiter plate was pre-coated with a monoclonal antibody specific for the NH2-terminus region of human Aβ42. Protein concentrations were determined using the bicinchoninic acid method (Sigma Aldrich, Deisenhofen, Germany). Samples were diluted with Aβ42 ELISA reaction buffer (i.e. 0.2 g/L KCl: 0.2 g/L KH_2_PO_4_, 8.0 g/L NaCl, 1.150 g/L Na_2_PO_4_, 5% BSA, 0.03% Tween-20, 1 x protease inhibitor) at pH 7.4, in order to investigate to samples at the same total protein concentration. Fifty μl of Aβ peptide standards or samples were then incubated with 50 μl rabbit anti human Aβ42 detection antibody, specific for the COOH-terminus of the 1–42 Aβ sequence, by shaking for 3 h at room temperature. After washing, detection was performed using 100 μl anti rabbit IgG HRP solution followed by 100 μl stabilized Chromogen. After adding 100 μl stop solution optical density was measured at 450 nm and Aβ 42 concentrations was calculated using a serial dilution of the Aβ peptide standard.

### Lung tissue analysis

Following sacrificing of the animals lungs were carefully removed and intratracheally fixed in 10% PBS-buffered Formalin (pH 7.4) at 4 °C for a minimum of 24 h. The dehydration occurred in a series of ethanol followed by a transfer into xylene. Lungs were then embedded in paraffin, sections were cut using a sliding microtome and shifted on Superfrost Ultra Plus object slides and dried over night at 40 °C. Afterwards slices were deparaffinised in xylene, followed by a rehydration in a series of ethanol (100%, 96%, 70%) and stained using haematoxylin-eosin (HE) for light microscopical evaluation.

### Bio-Plex cytokine assay

Systemic inflammation was assessed by measurement of the concentrations of interleukin (IL)-1α, IL-1β, IL-6, IL-17, keratinocyte chemoattractant/interleukin-8 (KC/IL-8), macrophage inflammatory protein (MIP)-1α, MIP-1β, regulated on activation, normal T cell expressed and secreted (RANTES), monocyte chemoattractant protein-1 (MCP-1), granulocyte colony-stimulating factor (G-CSF) and granulocyte-macrophage colony-stimulating factor (GM-CSF), in mouse plasma using a Bio-Plex murine cytokine platform. The assay was performed according to manufacturer’s instructions, as described previously [41]. Data were analysed with a Luminex 100 IS 2.3 system coupled to Bio-Plex Manager 4.1.1 software.

### Statistical analyses

In the study design, the foreseen number of mice per treatment group (i.e. 10 per group for WT mice, 16 per group for 5XFAD mice) was based on power calculations, with the assumption that the DEE exposure would accelerate the progressive phenotype (i.e. plaques, behaviour effects) of the 5XFAD mice [36] by 3 months. During the actual inhalation experiments, due to technical problems two mice were excluded from the analyses. Combined with the genotyping verification, this resulted in the following animal numbers per group: WT 3 weeks clean air exposed (*n* = 12); WT 3 weeks DEE (*n* = 10); WT 13 weeks air (*n* = 10); WT 13 weeks DEE (*n* = 8); 5XFAD 3 weeks air (*n* = 14); 5XFAD 3 weeks DEE (*n* = 14); 5XFAD 13 weeks air (*n* = 16); 5XFAD 13 weeks DEE (*n* = 18). Data were analysed using IBM-SPSS (version 22) and are expressed as mean ± SEM unless stated otherwise. Data that were obtained for both 5XFAD and WT mice were evaluated by one-way analysis of variance (ANOVA) with post-hoc analysis according to Tukey’s method. Possible interactions between genetic background and the DEE exposure were explored using additional 2-way ANOVA analysis. The 5XFAD-specific effect evaluations (i.e. effects of DEE exposure on amyloid plaque formation, whole brain Aβ42 protein levels and whole blood cytokine levels) were evaluated by Student’s t-test. Differences were considered statistically significant at *p* < 0.05.

## Results

### Effects of DEE inhalation exposure on body and organ weights of 5XFAD and WT mice

The weights of the mice were determined prior to the exposures as well as after the 3 and 13 weeks exposures. Results are shown in Table 2. There were no statistically significant differences in body weights after the 3 weeks exposure for the 5XFAD mice compared to the WT mice of the respective exposure groups. However, at this time point the DEE-exposed 5XFAD mice weighed significantly less than the clean-air exposed WT mice (ANOVA-Tukey, *p* < 0.001). This was also confirmed by evaluation of the body weight gain during exposure. The mean weight gain of the WT controls was 3.9 g whereas the DEE-exposed 5XFAD mice only gained 1.9 g on average (see Table 2; ANOVA-Tukey, *p* = 0.012). Body weights did not significantly differ before the start of the inhalations. These findings suggests that the combination of the 3 weeks exposure to DEE and the genetic background has an adverse impact on weight gain for the mice at this developmental stage. A two-way ANOVA analyses revealed that there was no interaction between both factors (F = 0.003, *p* > 0.1). For the 13 weeks exposure groups, there was no influence of exposure, genotype or a combination of both factors on body weight. This suggests that the effect observed after the 3 weeks exposure is transient.

**Table 2:** Body and organ weights of the 5XFAD mice and their WT littermates after 3 or 13 weeks exposure to DEE of clean air

	3 weeks study				13 weeks study			
	Air		DEE		Air		DEE	
	WT	5XFAD	WT	5XFAD	WT	5XFAD	WT	5XFAD
Age (days)	101.6 ± 0.5	101.6 ± 0.5	101.5 ± 0.2	101.4 ± 0.1	171.0 ± 0.1	170.9 ± 0.2	170.6 ± 0.2	170.3 ± 0.1
Body weight, sacrifice (g)	23.5 ± 0.5	22.2 ± 0.4	21.9 ± 0.5	20.6 ± 0.3^a^	23.4 ± 0.7	23.6 ± 0.9	24.3 ± 0.7	23.6 ± 0.5
Body weight gain (g)^c^	3.9 ± 1.3	3.4 ± 1.0	2.6 ± 2.7	1.9 ± 0.8^b^	5.4 ± 0.6	4.7 ± 0.7	4.4 ± 0.3	4.9 ± 0.5
Liver/body weight (mg/g)	40.1 ± 3.37	45.69 ± 2.55	38.68 ± 1.66	43.24 ± 1.20	47.72 ± 4.40	43.13 ± 0.90	47.26 ± 1.99	44.20 ± 0.83
Lung/body weight (mg/g)	5.63 ± 0.14	5.64 ± 0.13	6.06 ± 0.52	5.86 ± 0.15	4.69 ± 1.04	6.48 ± 0.11	6.10 ± 023	6.09 ± 0.19
Heart/body weight (mg/g)	5.40 ± 0.24	5.28 ± 0.13	4.91 ± 0.21	5.35 ± 0.14	6.68 ± 0.36	5.81 ± 0.33	6.09 ± 0.24	6.11 ± 0.29
Kidney/body weight (mg/g)	11.31 ± 0.17	12.34 ± 0.29	12.39 ± 0.46	12.31 ± 0.19	10.98 ± 0.10	12.14 ± 0.33	12.33 ± 0.14	12.01 ± 0.30
Spleen/body weight (mg/g)	2.91 ± 0.13	2.85 ± 0.07	2.77 ± 0.09	3.01 ± 0.09	3.17 ± 0.22	3.74 ± 0.47	3.11 ± 0.09	3.26 ± 0.10

Data represent mean ± standard error. Statistical analysis was performed using ANOVA with Tukey post hoc evaluation

^a^versus clean air exposed WT mice (3 weeks), *p* < 0.01; ^b^versus clean air exposed WT mice (3 weeks), *p* < 0.05

^c^Body weight gain at time interval between exposure start and sacrifice

The weights of liver, lung, heart, kidneys and spleen of the mice were determined at sacrifice (see Table 2). No statistically significant differences in weights of these organs were found after the 3 and 13 weeks exposures. Thus, neither the exposure to the DEE, nor the genetic background had an influence on the weights of these organs.

### Effects of DEE inhalation exposure on behaviour in 5XFAD and WT mice

**Fig. 2:**
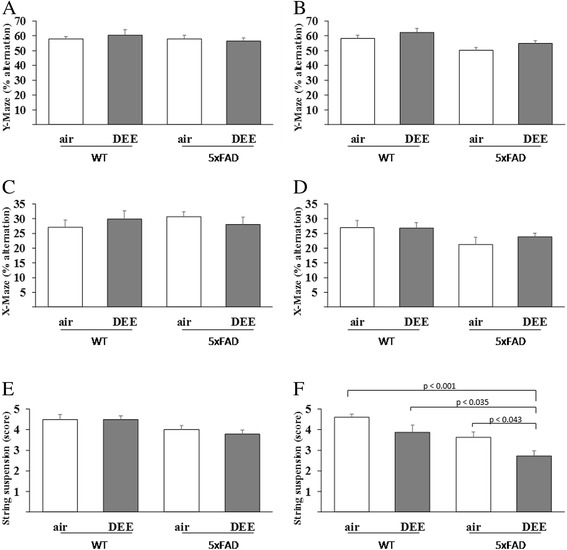
Behaviour tasks performances of WT and 5XFAD mice following inhalation exposure to DEE or clean air. Data represent mean ± standard error of the % alternation in the Y-maze task (panels **a** and **b**), % alternation in the X-maze task (**c** and **d**) and the string suspension task score (**e** and **f**) following 3 weeks exposure (**a**, **c** and **e**) or 13 weeks exposure (**b**, **d** and **f**) to clean air or DEE as indicated in the figures

The Y-maze and X-maze tasks were applied in this study to evaluate spatial working memory effects of the mice in association with the DEE exposure and genetic background. The string suspension task was performed to assess for grip strength and motor coordination. Results of these mouse behaviour tests are shown in Fig. 2. The total number of arm entries recorded during the 10 min interval for the Y-maze and X-maze tasks are shown in Table 3. After the 3 weeks exposure there were no statistically significant differences in spatial working memory between the four groups, as revealed from outcomes of the Y-maze (see Fig. 2a) and X-maze tasks (panel c). The total number of arm entries also did not differ significantly between the treatment groups indicating overall similar explorative behaviour for both maze tasks (Table 3). There were also no statistically significant differences in the string suspension task (Fig. 2e). Irrespective of the genetic background of the mice, the 3 weeks inhalation exposure to DEE did not affect the performance in any of the three behaviour tasks.

**Table 3:** Total number of arm entries in Y-maze and X-maze tasks

	3 weeks study				13 weeks study			
	Air		DEE		Air		DEE	
	WT *n* = 12	5XFAD *n* = 14	WT *n* = 10	5XFAD *n* = 14	WT *n* = 10	5XFAD *n* = 16	WT *n* = 8	5XFAD *n* = 18
Y-maze number of entries	41.5 ± 2.8	37.6 ± 3.2	39.0 ± 4.0	33.8 ± 2.8	46.2 ± 4.9	56.5 ± 3.5	51.3 ± 4.8	44.6 ± 3.9
X-maze number of entries	52.9 ± 3.9	47.6 ± 4.3	53.1 ± 5.8	44.4 ± 3.6	61.1 ± 4.4	77.2 ± 10.0	63.6 ± 5.6	56.1 ± 3.9

Data represent mean ± standard error. Statistical analysis was performed using ANOVA with Tukey post hoc evaluation

Also in the 13-week study, there were no statistically significant differences in performance in spatial working memory tasks (see Fig. 2b and d for the Y-maze and X-maze task, respectively). For this time point, also the total number of arm entries did not differ in the Y-maze and X-maze task (Table 3), thus revealing similar explorative behaviour. Taken together, the sub-chronic (i.e. 13 week) inhalation study did not reveal any major adverse effect of the DEE on spatial working memory.

In contrast, with the string suspension task clear differences were found following the 13 weeks inhalation (see Fig. 2f). Both the genetic background and the DEE exposure affected the performance of the mice in this motor function assessment test. The 5XFAD mice that were exposed for 13 weeks to DEE performed significantly less than all three other groups (ANOVA-Tukey, *p* < 0.001 vs the clean-air exposed WT mice, *p* = 0.035 versus the DEE exposed WT mice and *p* = 0.043 vs the air exposed 5xFAD mice). A two-way ANOVA analysis revealed no significant background-by-exposure interaction (F = 0.099). Most importantly, the observed difference between the DEE-exposed 5XFAD mice and the clean air exposed 5XFAD littermates demonstrates that sub-chronic inhalation exposure to DEE aggravates the motor function phenotype of this transgenic mouse model of AD. Interestingly, the WT mice exposed to DEE also tended to perform less well in the string suspension task than the clean air-WT littermates, but this effect did not reach statistical difference (*p* = 0.071).

### Effects of DEE inhalation exposure on Aβ plaque formation in the brains of 5XFAD mice

**Fig. 3:**
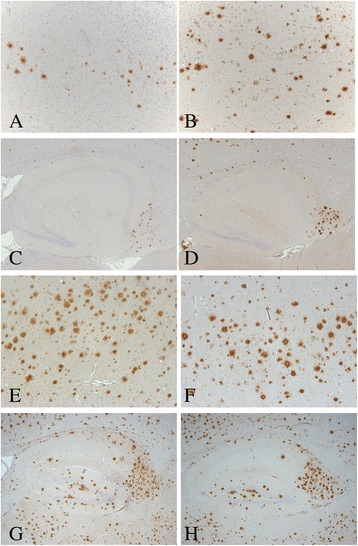
Representative images showing amyloid-β plaque staining in cortex (**a**, **b**, **e** and **f**) and hippocampus (**c**, **d**, **g** and **h**) of brains sections from 5XFAD mice. The accumulation of Aβ42 (*reddish-brown* colour) was localised by immunohistochemistry in sections of paraffin-embedded brain hemispheres. Hippocampus (50 x magnification) and cortex (100 x magnification) from the same animal are shown for each time point and exposure. i.e. 3 weeks to clean air (**a** and **c**), 3 weeks to DEE (**b** and **d**), 13 weeks to clean air (**e** and **g**) and 13 weeks to DEE (**f** and **h**), respectively

**Fig. 4:**
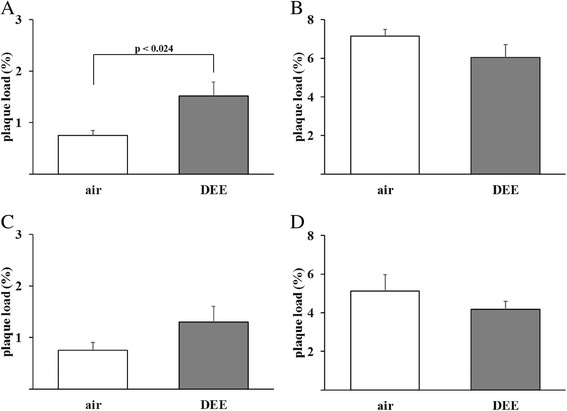
Plaque load in cortex (**a**, **b**) and hippocampus (**c**, **d**) of 5XFAD mice following 3 weeks (**a**, **c**) or 13 weeks (**b**, **d**) exposure to DEE or clean air. Quantitative Aβ42 plaque analyses were performed via calculation of the % of total plaque load in the analysed area of the section (*n* = 11–16 mice per group)

To evaluate the impact of the DEE exposure on plaque formation in 5XFAD mouse model parasagittal brain slices were stained with an antibody against Aβ42 and quantitative plaque analysis was performed with imaging software. Representative light microscopical pictures from cortex and hippocampus of 5XFAD brain slices stained for Aβ42 are shown in Fig. 3. The brains of selected WT animals were also stained, but no plaques could be detected (data not shown). Results of the plaque analyses are shown in Fig. 4. After the 3 weeks exposure, the level of plaque formation was found to differ between the DEE and the control group (See Fig. 4a and c). For the cortex region, there was a statistically significant difference in plaque formation between the DEE and clean-air exposed mice, Student’s t-test, *p* = 0.024). For the hippocampus, the difference did not reach statistical significance. These data indicate that the inhalation exposure to DEE causes accelerated plaque formation in the 5XFAD mouse model. After the sub-chronic exposure (Fig. 4b and d) no statistically significant differences were observed. After the 13 weeks exposures, plaque density in cortex as well as hippocampus was by far more pronounced than after the 3 weeks exposure.

Since the 3 weeks DEE-exposed 5XFAD mice gained significantly less weight than the clean-air exposed WT littermates (see Table 2), the relation between body weight and plaque load was further explored in the 5XFAD animals. A Pearson correlation analysis revealed a significant inverse association between body weight and plaque load in cortex (*r* = −0.467, *p* < 0.05), but not in hippocampus (*r* = −0.123).

### Effects of DEE inhalation exposure on human Aβ42 protein levels in 5XFAD mice

**Fig. 5:**
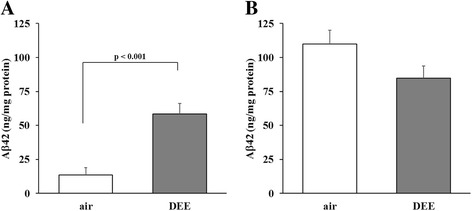
Amyloid-β protein levels in mouse brain homogenates. Human Aβ42 protein levels were determined by ELISA following 3 (**a**) or 13 weeks (**b**) exposure to DEE or clean air (*n* = 5–8 mice per group)

Human Aβ42 protein levels were determined in brain homogenates of the 5XFAD mice by ELISA. The applied ELISA method allowed for the detection of both soluble and insoluble Aβ42 fragments. Results are shown in Fig. 5. In line with the findings on plaque formation, mice that were exposed to DEE for 3 weeks showed markedly higher Aβ42 protein levels than the control mice (Student’s t-test, *p* < 0.001). For this time point, the whole brain Aβ42 protein levels were significantly correlated with plaque load in cortex (Pearson *r* = 0.710, *p* = 0.014) as well as hippocampus (*r* = 0.608, *p* = 0.047). The 13 weeks inhalation study revealed no significant differences in Aβ42 levels between the DEE and clean air control littermates.

### Pulmonary histology and systemic inflammation

**Fig. 6:**
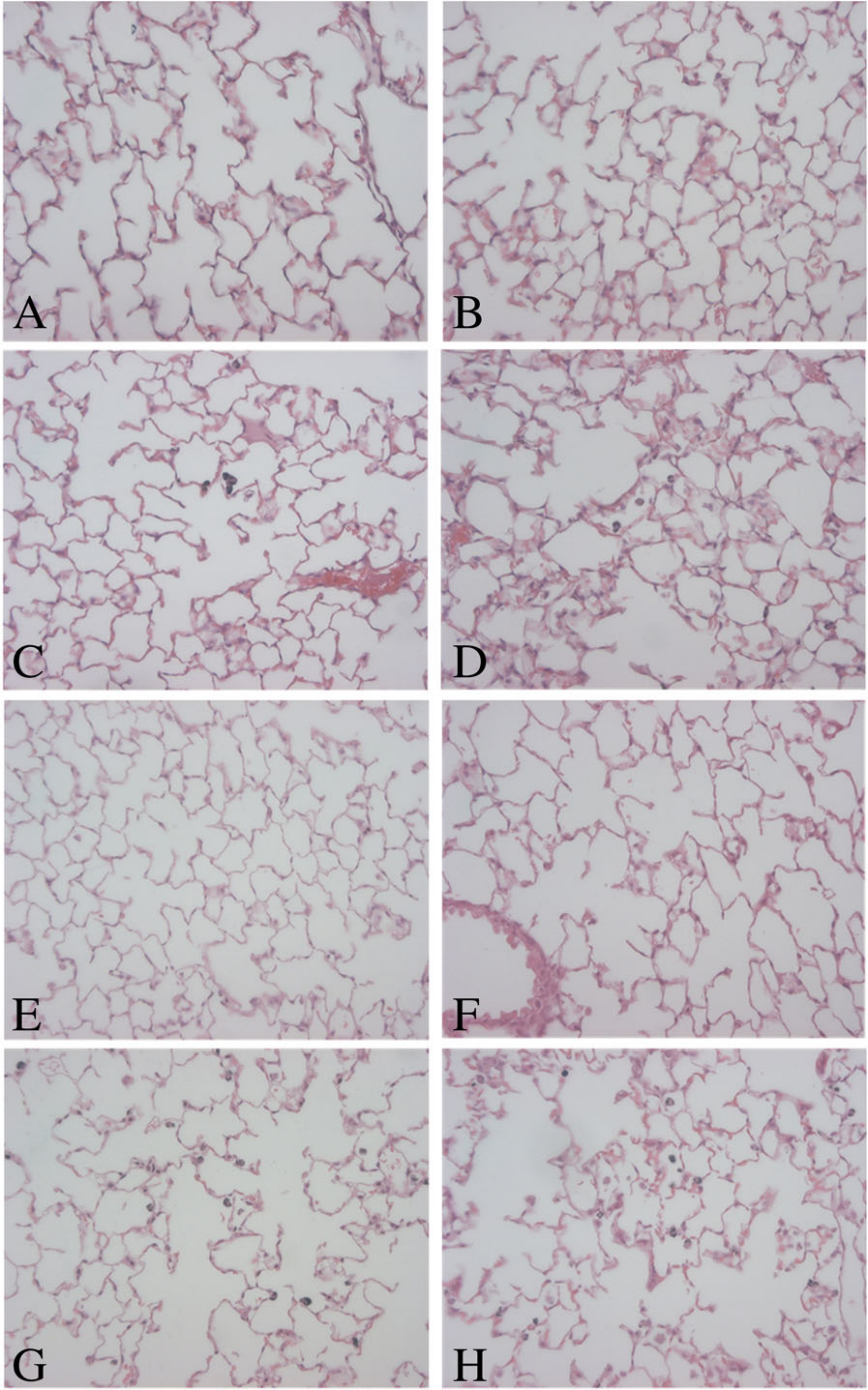
Representative images of haematoxylin-eosin stained mouse lungs. Tissue sections shown are from: **a** 3 weeks clean air-exposed 5XFAD mouse; **b** 3 weeks clean air-exposed WT mouse; **c** 3 weeks DEE-exposed FAD mouse; **d** 3 weeks DEE-exposed WT mouse; **e** 13 weeks clean air-exposed 5XFAD mouse; **f** 13 weeks clean air-exposed WT mouse; **g** 13 weeks DEE-exposed 5XFAD mouse; **h** 13 weeks DEE-exposed WT mouse. Original magnification 640×

To evaluate the pulmonary effects of the DEE exposure in the 5XFAD mice and WT littermates, lung sections were analysed. Representative pictures of HE-stained tissues are shown in Fig. 6. Histological evaluation of the mice lungs revealed no obvious differences in tissue structure related to DEE exposure or genetic background. The lungs of 5XFAD mice were also found to be comparable to those of their WT littermates. Deposition of clustered diesel exhaust particles could be clearly detected in the lungs of the DEE-exposed mice (see Fig. 6). However, these depositions were not associated with any obvious development of structural irreversible changes within the pulmonary tissues of the WT and 5XFAD mice.

**Fig. 7:**
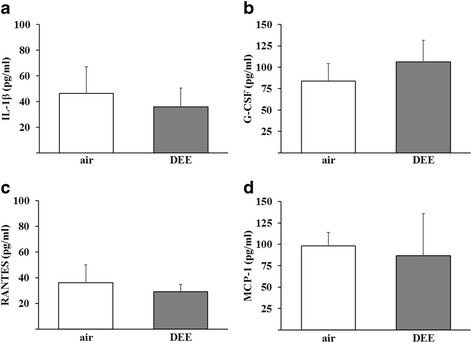
Plasma levels of IL-1β (panel **a**), G-CSF (**b**), RANTES (**c**) and MCP-1 (**d**) in blood of mice following 13 weeks exposure to DEE or clean air. Data were obtained by Bio-Plex murine cytokine platform from blood collected at sacrifice (*n* = 5 mice per group)

A Bio-Plex murine cytokine platform was used to evaluate the levels of cytokines in the blood of DEE and clean air-exposed 5XFAD mice as an indicator of systemic inflammation. Results are shown in Fig. 7. The levels of the four cytokines that could be readily detected in the blood of the mice (i.e. IL-1β, RANTES, G-CSF and MCP-1) did not significantly differ between the DEE and sham exposure conditions. The levels of IL-1α, IL-6, IL-17, KC, GM-CSF, MIP-1α and MIP-1β were not consistently above detection limit. These findings indicated that the DEE inhalation exposure regime did not induce a sustained systemic inflammatory response.

## Discussion

In the present study, we demonstrated that inhalation exposure to DEE leads to accelerated formation of Aβ-plaques as well as motor function impairment in 5XFAD mice. Studies in transgenic mouse models of AD, including the 5XFAD model, have contributed to the understanding of mechanisms involved in β-amyloid pathology [42–44]. The 5XFAD model has also been used to assess potential beneficial aspects of candidate-drugs or animal housing enrichment [45–48]. Factors that could accelerate AD-like pathology, like environmental toxicants or stress have been much less topic of investigation. Devi et al. [37] showed increased Aβ42 levels and accelerated Aβ plaque formation in the hippocampus of 3 months old female 5XFAD mice due to restraint stress, induced by placing them in plastic tubes for 5 days (6 h/day). In our study, accelerated plaque load and increased whole brain Aβ42 protein levels were detected in 15 weeks old female 5XFAD mice following 3 weeks DEE inhalation exposure. The results from Devi et al. [37] indicate that stress may also contribute to CNS pathologies in rodent inhalation studies, specifically in nose-only inhalation protocols, as this requires restraint of the animals. In the present study, a whole body inhalation approach was used with simultaneous exposure of littermates to DEE and clean air at otherwise identical conditions. Indeed, the specific caging, handling and treatment conditions of the 5XFAD mice may have led to differences in Aβ42 protein levels and extent of plaque load, when compared to other studies with similar-aged 5XFAD mice. However, with our study design it can be assured that the observed effects in this AD mouse model were driven by the DEE inhalation per se.

Several other investigators have reported findings that support a role of DEE inhalation in AD pathogenesis. Levesque et al. [8] showed increased Aβ42 levels in frontal lobe of male Fischer 344 rats after 6 months exposure to DEE. Increased Aβ42 levels were also reported in a 90-day rat inhalation study with resuspended diesel exhaust particles, albeit at very high concentrations (>500 mg/m^3^) [49]. Bhatt et al. [29] showed increased levels of Aβ40 and β-secretase in C57BL/6 mice following 9 months exposure to concentrated PM_2.5_. To the best of our knowledge, the results of our study are the first to show an effect of DEE inhalation on actual Aβ plaque formation in mouse brain, and thus support the initial studies that suggested the link between ambient air pollution and Aβ plaque pathology in humans [26, 27].

Notably, in our study cortical plaque load and whole brain Aβ42 protein levels were inversely correlated with body weight following the 3 weeks exposures. This would suggest that the accelerated plaque formation could be the cause of, or the result of, impaired body weight gain in these young DEE-exposed 5XFAD mice. The absence of such associations in the 13 weeks exposed animals indicates the transient nature of this phenomenon. Significant lower body weights for the 5XFAD mice compared to their WT littermates were previously found starting at an age of 9 months [36]. We consider it unlikely that the amyloidogenic effect of the DEE is a mere indirect effect resulting from a direct adverse impact of DEE on body weight. Absence of effects of DEE inhalation on body weight has been demonstrated for inhalation studies in rats as well as mice at exposure concentrations that were similar to those in present study [50, 51]. Our data rather suggest that the weight changes are a secondary effect, resulting from a primary effect of DEE on plaque formation. However, a combined contribution of DEE and the 5XFAD background cannot be ruled out.

In the 13 weeks exposed 5XFAD mice, significant differences in plaque formation and Aβ42 protein levels were no longer observed. Levels of Aβ42 have been demonstrated to rise drastically in brain of 5XFAD mice within the first 6 months of age [34]. The marked differences in Aβ42 levels in the air-exposed (i.e. control) 5XFAD mice of different age (Fig. 5a versus b) confirm the rapid plaque development phenotype in this transgenic model. We therefore believe that the aggressive pathology of the 5XFAD model in the 13 weeks exposed mice saturated the early plaque-accelerating effect of the DEE. In line with this, recently, a long-term (i.e. 11 months) environmental enrichment was found to improve motor performance in 5XFAD mice, whereas this treatment failed to benefit working memory performance, anxiety and Aβ plaque load [47]. It was concluded that the relative fast and aggressive pathology of the 5XFAD model obscures interventions that are beneficial to disease progression [47]. Our study results indicate that this is also true for interventions that worsen disease hallmarks.

To assess effects of DEE inhalation exposure on cognition, Y-maze and X-maze tasks were performed, whereas motor function was evaluated by the string suspension. Importantly in this regard, all animals (i.e. including the clean air exposed “control” mice) were placed in the inhalation units with 2 animals per cage. Moreover, in concordance with conventional inhalation toxicology protocols, the mice were housed without inverted light conditions and exposed during daytime, i.e. during their sleep phase. Hence, also the post-exposure behaviour tasks were performed during daytime, while such testing is considered more suitable during the active phase for these night-active species [52]. These and further factors may very well have influenced the age of onset and extent of behaviour phenotype as reported for the 5XFAD mice in other studies. We did not observe significant behaviour impairments following the 3 weeks DEE exposure. Furthermore, no differences between the 5XFAD and WT littermates of the respective exposure groups were seen at this young-age (i.e. < 3 months) time point. The latter agrees with the pattern of age-dependent phenotype development of this transgenic model [34, 36, 44]. The 13 weeks DEE-exposure also did not cause statistically significant differences in the Y-maze and X-maze tasks. However, irrespective of the exposure condition the 5XFAD animals tended to show a slightly lower spatial working memory performance than the WT animals (see Fig. 2b and d for the Y-maze and X-maze task, respectively). Effects of age-differences among the different treatment groups could be excluded because of the littermate-study design.

However, significant effects of DEE on the performance in the string suspension task were found following the 13 weeks exposures. Thus, at this time point, an adverse impact on motor function was identified while differences in Aβ42 levels and plaque load were no longer present. As already mentioned, environmental enrichment of 5XFAD mice revealed a similar contrast [47]. In our hands, the DEE-exposed 5XFAD animals also performed less than their DEE-exposed WT littermates as well as the clean air-exposed WT littermates. Thus, apart from a specific effect of the DEE there also appeared to be a contribution of the transgenic background on motor function loss. Age-dependent performance impairment in the string suspension task by female 5XFAD mice has been described previously [36].

In our study, DEE inhalation exposure also tended to affect motor performance in the WT mice. Although the effect was not statistically significant, it suggests that DEE could cause motor impairments in other mouse strains. Indeed, others have shown locomotive activity effects in rats following exposure to DEE (6 mg/m^3^, 8 h/day for 16 weeks) [53] or mice exposed to resuspended diesel exhaust particles (72 mg/m^3^, 90 min/day for 4 days) [54]. Interestingly, decreased locomotor activity has also been shown in mice following prenatal exposure [55]. Sensory-motor impairments may occur due to axonal defects and these have been observed in studies with AD patients [56]. Axonopathy and deficits like swelling of accumulated motor proteins, organelles and vesicles have been detected in an APP transgenic mouse model carrying the Swedish mutation [57]. It has been demonstrated that 5XFAD mice develop, in addition to working memory deficits, an age-dependent motor phenotype. The motor phenotype correlates with spinal cord pathology, as demonstrated by abundant intraneuronal Aβ accumulation and extracellular plaque deposition [36].

The underlying molecular mechanisms of DEE-induced accelerated Aβ plaque deposition and motor impairment remain to be clarified. Two major principle pathways have been discussed whereby ultrafine air pollution particles may induce adverse effect in the CNS, namely a direct pathway whereby the particles physically enter the brain parenchyma, and an indirect pathway whereby peripheral effects contribute to neurotoxicity [19, 58]. Inhalation studies with PM and DEE have linked oxidative stress and associated induction of inflammation to pulmonary as well as cardiovascular effects [1, 59]. Systemic inflammation has been discussed to be involved in AD pathogenesis [31–33] and could thus provide a mechanism whereby inhaled DEE could affect the CNS indirectly. In our hands, the diluted DEE inhalations at a particle dose of 0.95 mg/m^3^ did not cause notable irreversible pathological changes in the lungs of the mice of both backgrounds. The profile of blood inflammatory markers also did not differ between DEE and clean air-exposed mice. Taken together, our findings suggest that observed amyloidogenesis and motor function impairment effects were not mediated by strong pulmonary toxicity and/or systemic inflammatory responses. However, it should be emphasized that these analyses were performed 11 days after the last exposure (see also Fig. 1), and thus we cannot rule out the possible occurrence of mild and transient inflammatory effects during the DEE inhalations.

Induction of local oxidative stress and inflammation following direct translocation of the component of ultrafine carbonaceous particles of DEE into the brain represents the major alternative or complementary mechanism of action [19]. Inflammation and oxidative stress has been shown to trigger β-amyloid pathology (for review see [60, 61]), but brain tissue expression analyses of markers of these processes was not pursued in our study, in view of the considerable time interval between the last days of DEE exposure and tissue collection. However, brain-region specific inflammatory and oxidative stress responses have been demonstrated previously in a considerable number of rodent inhalation studies with PM ([2–4, 62] and DEE [5, 6, 8, 9, 63]. Translocation into the brain was firstly shown in a rat inhalation study for ultrafine ^13^C particles [64], and recently regained major attention in a study were magnetite particles of proposed external origin were shown in human brains [65].

However, the potential contribution of non-particulate components within the DEE pollutant mixture should also be considered in our study. Apart from the carbonaceous particles and components (metals, organics) absorbed on their core, the exhaust of diesel also contains various non-particulate compounds including CO, NO_x_, SO_2_, and volatile organic compounds. The levels of the prevailing gases CO and NOx in the diluted DEE in the exposure units were of an order of magnitude at which adverse effects have been found to be absent (and even neuroprotective) in mice [66]. Findings from intratracheal instillation studies with diesel exhaust particles [7] or inhalation studies with resuspended particulates [49, 54] are in strong support for a role of the particle component of the DEE, although dosimetry aspects should be taken into account here. However, the effects observed in our present study could also have resulted from interactions among the various phases. This aspect has been elegantly addressed in a recent study by Tyler et al. [67] through the parallel testing of fine or ultrafine particles generated from gasoline or diesel emissions in either presence or absence of gaseous compounds. Interestingly, neuroinflammatory effects (i.e. cytokine expression in hippocampus) tended to be the strongest in the animals that were exposed to combination of ultrafine PM fraction and the gaseous components. The authors concluded their findings supported the observed epidemiological associations between roadway proximity and neurological outcomes, indicative of a dominant role for freshly generated ultrafine particles in the presence of combustion-derived gas phase pollutants [67].

## Conclusions

We showed that DEE inhalation causes accelerated formation of Aβ-plaques and motor function impairment in 5XFAD transgenic mice. Our study provides further support that the brain is a relevant target for the effects of inhaled DEE and suggest that long-term exposure to this ubiquitous air pollution mixture may promote the development of AD. Our results should be viewed in the context of the recently published population-based findings on road proximity and incident dementia [16]. Further research is needed to determine the relevance of the applied dosimetry and animal model for humans in relation to relevant exposure situations. Toxicological investigations on the contributions of specific particulate versus non-particulate components of motor vehicle exhaust and other traffic-related stress factors (e.g. noise) to AD pathogenesis represent a major challenge.
